# Late Jejunojejunal Perforation After Laparoscopic Roux-en-Y Gastric Bypass: A Systematic Narrative Review

**DOI:** 10.7759/cureus.100492

**Published:** 2025-12-31

**Authors:** Oday Al-Asadi, Karim Ataya, Almoutuz Aljaafreh, Farah Aldhaher, Mostafa Mahran

**Affiliations:** 1 General Surgery, Homerton University Hospital, London, GBR; 2 Surgery, Homerton University Hospital, London, GBR; 3 Upper Gastrointestinal Surgery, King’s College Hospital, London, GBR; 4 Radiology, Guy’s and St Thomas’ NHS Foundation Trust, London, GBR; 5 General and Upper Gastrointestinal Surgery, Ain Shams University, Cairo, EGY; 6 Upper Gastrointestinal Surgery, Homerton University Hospital, London, GBR

**Keywords:** anastomosis, jejunojejunal, jejuno-jejunal, jejunojejunal perforation, jejunum, laparoscopic roux-en-y gastric bypass, lrygb, perforation, roux-en-y gastric bypass, rygb

## Abstract

Late perforation of the jejunojejunal (JJ) anastomosis after laparoscopic Roux-en-Y gastric bypass (LRYGB) is an exceedingly uncommon but potentially life-threatening complication. The widely non-specific presentation and the vast range of possible causes can delay diagnosis and increase morbidity. In this study, we aim to integrate the results of published literature on late JJ perforation after LRYGB in terms of patients’ clinical characteristics, presentation, duration between the RYGB operation and the onset of JJ perforation, possible causes, as well as the outcome of the patients. We performed a comprehensive literature search of PubMed, CINAHL Plus, Embase, and EBSCOhost from inception to October 2025 using terms related to “Roux-en-Y gastric bypass,” “jejunojejunal,” “JJ,” and “perforation.” Case reports and case series describing late JJ perforation after LRYGB in adults were eligible. Two reviewers independently screened titles, abstracts, and full texts. Data were extracted on patient characteristics, time from index operation to perforation, predisposing factors, diagnostic tools, operative findings, and outcomes. Evidence was reviewed systematically and then narratively synthesised. A meta-analysis was not possible due to the case-based nature of the available literature. Eight studies reporting 12 patients with late JJ perforation following LRYGB were identified. Most patients were middle-aged females and presented months to years after surgery with severe, acute abdominal pain, with no specific localising signs. Radiological findings were variable and occasionally non-diagnostic. Proposed mechanisms included marginal ulcers, ischaemia, infection, stress, phytobezoar, and tumour implantation. All patients required operative management, most commonly laparoscopic suture repair or revision of the JJ anastomosis, with generally favourable outcomes with early treatment. Although late JJ perforation after LRYGB is exceptionally rare, it should be considered in any patient presenting with an acute abdomen with a past history of bariatric operation, irrespective of the interval since surgery. Early radiological assessment with cross-sectional studies, a low threshold for diagnostic laparoscopy, and urgent surgical intervention are crucial. Standardised reporting of future cases and collaborative registries are required to define incidence, risk factors, and best management strategies.

## Introduction and background

In the current practice in bariatric surgery, laparoscopic Roux-en-Y gastric bypass (LRYGB) is described as the gold standard procedure for weight loss due to its effective long-term results in treating obesity and its related diseases. It was first described by Wittgrove in 1994 [1]. The procedure comprises the formation of anastomosis between the stomach and the jejunum (GJ) with another anastomosis between the jejunum and jejunum (JJ) at two measured points of the bowel length. However, it carries several early and late complications related to these joints formation which can be manifested many years following the primary operation. These late complications might include obstruction, stricture, ulceration, or perforation. Although many studies focused on late GJ anastomotic perforation, there are limited studies published on late JJ perforation, which seems to be extremely rare (less than 1%) [2,3]. Perforations after 30 days from the index operation are regarded as late [3]. This type of perforation at the JJ joint is still poorly understood.

In this study, we aim to integrate the results of published literature on late JJ perforation after LRYGB in terms of patients’ clinical characteristics, presentation, duration between the RYGB operation and the onset of JJ perforation, possible causes, as well as the outcome of the patients. Due to its rarity and the lack of high-quality evidence in the literature, the available case reports and small case series were systematically reviewed but narratively synthesised. A meta-analysis was not possible.

## Review

Methodology

A comprehensive literature search was conducted across four electronic databases: CINAHL Plus with Full Text (n = 2), EBSCOhost (n = 1), Embase (n = 17), and Ovid MEDLINE (n = 21) from inception to 23rd of October 2025. No additional filters were applied. The search strategy combined Medical Subject Headings (MeSH) and free-text terms related to laparoscopic RYGB and JJ perforation, including “Laparoscopic Roux-en-y gastric bypass,” “LRYGB,” “Roux-en-Y gastric bypass,” “RYGB,” “jejunum,” “jejunojejunal,” “jejuno-jejunal,” “J-J,” “JJ,” “perforation,” “perforat,” and “anastomosis.” The reference lists of included articles were also manually screened to identify additional studies. Study selection outcomes and extracted data are summarised in Table 1.

**Table 1:** Inclusion and exclusion criteria. LRYGB = laparoscopic Roux-en-Y gastric bypass; JJ = jejunojejunal

Inclusion criteria	Exclusion criteria
Adults >18 years old who had previous LRYGB	Studies irrelevant to the question of the review (late JJ perforation after LRYGB)
studies reporting late (>30 days after surgery) perforation at the JJ anastomosis	Late perforation at the gastrojejunostomy
Case reports, case series, and relevant reviews	Studies on other JJ-related complications, e.g., intussusception
Clinical presentation, diagnostic imaging, operative findings, management, or patient outcomes	Non-English articles
Publications in the English language	Duplicate studies
Full-text articles	

**Figure 1:**
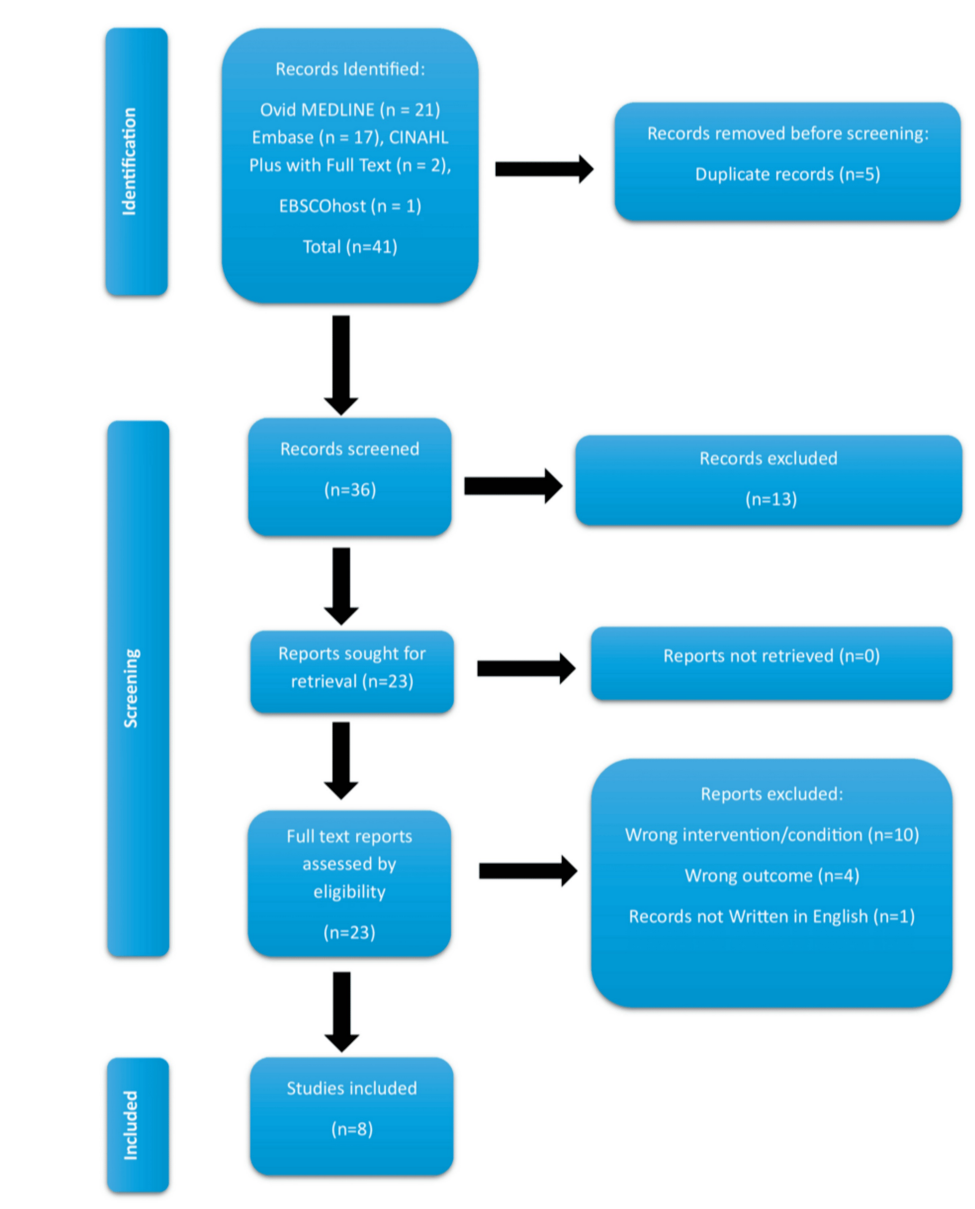
Preferred Reporting Items for Systematic Reviews and Meta-Analyses (PRISMA) flow diagram.

The study followed the Preferred Reporting Items for Systematic Reviews and Meta-Analyses (PRISMA) guidelines while conducting this research. Initial search, as well as the search of references, yielded 41 studies. Following the removal of duplicates, a review of titles and abstracts of 36 studies was conducted. Overall, 13 records were irrelevant to the study question. Another 10 studies discussing GJ, not JJ, perforation (wrong intervention/condition) and four studies related to JJ intussusception (wrong outcome) were removed from the search. One study was not published in the English language. The remaining eight full-text studies met the inclusion criteria for the qualitative synthesis. The PRISMA flow chart presented in Figure 1 summarises the search method.

Study Selection

The literature set provided was reviewed by two authors (OA and KA) independently, who checked the relevance of each title and abstract, and full texts if available. Differences in interpretation were resolved through discussion. A third reviewer (AA) offered input when needed to maintain consistency and decrease possible selection bias.

Quality Assessment

All studies included were either small case series or case reports and were therefore classified as Level 4 evidence according to the Oxford Centre for Evidence-Based Medicine (CEBM) 2009 hierarchy (Table 2).

**Table 2:** Study designs and level of evidence. *: Evidence levels per Oxford Centre for Evidence-Based Medicine 2009: 4 (case series/case reports).

Authors	Journal	Study type	Evidence level*	Number of patients
Sammut et al. [4]	Annals of the Royal College of Surgeons of England	Case report	4	1
Al Kandari et al. [5]	International Journal of Surgery Open	Case report	4	1
Elhardello et al. [1]	Obesity Surgery	Case report	4	1
De Hous et al. [6]	Acta Chirurgica Belgica	Case report	4	1
Gonzalez-Pezzat et al. [7]	Obesity Surgery	Case report	4	1
Kröll et al. [8]	Obesity Surgery	Case report	4	1
Goitein et al. [2]	Obesity Surgery	case series	4	3
Kalaiselvan et al. [3]	Surgery for Obesity and Related Diseases	Case series	4	3

Data Extraction

A standardised extraction sheet was used. Data collected included patient demographics, interval between LRYGB and perforation, presenting symptoms, laboratory and imaging findings, intraoperative findings, suspected underlying mechanism, type of surgical intervention, and postoperative outcomes. Extraction was performed independently by two reviewers, and discrepancies were resolved through discussion and agreement.

Data Synthesis

Given the heterogeneity in study design, reporting quality, and patient characteristics, a narrative synthesis approach was adopted. The Joanna Briggs Institute (JBI) Risk of Bias Assessment tool was used for the same reason. Findings were organised thematically into clinical presentation, diagnostic imaging, intraoperative findings, underlying mechanisms, and management strategies. A summary table was created to compare key variables across all reported cases, as presented in Table 3.

**Table 3:** Analytic synthesis of available studies. LRYGB = laparoscopic Roux-en-Y gastric bypass; JJ = jejunojejunal

Authors	Age (y)	Sex	Time since LRYGB	Presentation	Imaging	Finding at surgery	Management	Outcome	Possible mechanism
Sammut et al. [4]	40	Female	18 months	Abdominal pain for 10 hours	Free air on X-ray	Large JJ perforation	Suture repair with omental patch	Discharge on day nine	Phytobezoar
Al Kandari et al. [5]	46	Female	15 weeks	Abdominal pain for two days	Free air on X-ray	1.5 cm anterior JJ perforation	Suture repair with omental patch	No complication	Not clear (recent laparoscopic appendicectomy)
Elhardello et al. [1]	48	Female	9 years	Severe abdominal pain with diarrhoea	CT showed JJ perforation	Hole at recess/blind end of JJ anastomosis	Stapled resection of the blind end	Discharged on day seven	Histopathology confirmed ischaemic ulcer
De Hous et al, [6]	59	Female	5 years	Acute abdominal pain	CT perforation at JJ	Perforated mass at JJ towards the BPL side	Resection with refashioning of the JJ	Discharged on day six	Histopathology confirmed malignant melanoma
Gonzalez-Pezzat et al. [7]	38	Male	6 months	Colicky pain and early satiety for over five days	LUQ abscess on CT	-	Laparotomy, resection, and re-anastomosis of JJ	No complications	No clear cause
Kröll et al. [8]	36	Female	11 months	Severe crampy abdominal pain and diarrhoea	Free air on CT	5 mm JJ perforation at the mesenteric side	Laparotomy with excision of the perforation margin and primary closure	No complications	No clear cause
Goitein et al. [2]	45	Female	8 weeks	Acute abdominal pain with peritoneal signs	Normal X-ray and contrast	Pinhole JJ perforation	Laparoscopic closure with interrupted sutures	Recovered well	No clear cause
	47	Female	8 weeks	Acute abdominal pain with peritoneal signs	Free air on X-ray	2 mm JJ perforation	Laparoscopic closure with interrupted sutures	Recovered well	No clear cause
	48	Female	7 weeks	Acute abdominal pain with peritoneal signs	Free air on X-ray	JJ perforation with phytobezoar	Laparotomy defect closed with interrupted sutures	Recovered well	Phytobezoar
Kalaiselvan et al. [3]	25	Female	18 weeks	Acute abdominal pain	CT showed perforation	Perforation at the JJ anastomosis	Excision with re-anastomosis	Discharged after three weeks	Not clear (laparoscopic cholecystectomy two weeks before)
	44	Female	7 weeks	Abdominal pain	CT showed perforation	Perforation at the JJ anastomosis	Disconnected JJ with two separate (alimentary and biliary stomas), then re-anastomosis 16.5 months later	Discharged on day 18 after the last operation	Not clear (undue emotional stress)
	47	Female	9 weeks	Central acute abdominal pain	CT showed contrast leak at JJ	3 mm posterior JJ defect	Creation of a controlled fistula with an 8-F Foley catheter, which was removed after four weeks	Represented 42 days later with septic shock, possibly due to reperforation and died	Not clear (continued to have aspirin against advice)

Results

Thematic Synthesis

The available evidence on late JJ perforation following LRYGB is extremely scarce. They are mostly presented as individual case reports or short case series, which prevents useful quantitative analysis. A total of 12 patients with late JJ perforation following LRYGB were identified across eight published studies. Most patients, 11/12 (91.6%), were female, and the reported age range was between 25 and 59 (mean = 44) years.

The time interval between the index LRYGB and the development of JJ perforation varied considerably, reported as early as seven weeks and as late as nine years after surgery [1,3]. This wide time range indicates that this complication can happen at any time following the initial surgery.

Unlike GJ perforation, the majority of case reports did not show any significant history of smoking or positive tests for *Helicobacter pylori *infection. However, they cannot be completely excluded as known possible risk factors from these small studies.

Regarding clinical presentation, apart from one case report with gradual pain over five days [7], a short onset, acute abdominal pain within one to two days was the most consistent symptom. Almost all patients exhibited signs of peritonism, pyrexia, tachycardia, or systemic inflammatory response at the time of admission, and several had established sepsis. Laboratory tests commonly showed raised white cell counts and C-reactive protein levels, supporting the underlying inflammatory or infective process.

Preoperative imaging, including plain films and CT, was performed in most cases and frequently showed pneumoperitoneum and free intraperitoneal fluid. One case showed a localised intra-abdominal abscess [7], and contrast extravasation was evident in another patient [3]. In several reports, CT suggested a perforation involving the JJ anastomosis; however, the precise underlying mechanism was often not definitively identified until surgical exploration. Intraoperative findings demonstrated a wide range of aetiologies. Reported mechanisms included stress ulceration or ischaemic injury [1,3,8], mechanical obstruction due to a large phytobezoar [2,4], and recent surgery [3,5]. Additional causes, including abscess-associated perforation and metastatic infiltration from malignant melanoma, were noticed [6,7]. An infective cause or change in the gut flora after LRYGB was also raised as a possible aetiology [4]. Idiopathic perforations, where no clear precipitating factor was found, were frequently noticed [2-4,7].

All patients underwent surgical exploration, 4/12 (33.3%) with laparoscopy and 8/12 (66.6%) with laparotomy. Most operations were initially started laparoscopically, although conversion to laparotomy was often required in the presence of contamination, dense adhesions, or the need for bowel resection. Reported interventions were variable. They included primary closure of the JJ ulcer or defect, with or without Graham patch; segmental small-bowel resection with joint-refashioning anastomosis; creation of a controlled fistula using a Foley catheter; and temporary diverting jejunostomy in a systemically unwell patient, requiring staged reconstruction. The outcomes were generally favourable, with the majority of patients recovering well without immediate or long-term complications. However, one mortality case was reported in the Kalaiselvan et al. cohort who presented with recurrent sepsis due to presumed recurrent perforation several weeks after initial management with controlled tube fistulation [3].

Risk of Bias Assessment

The overall JBI quality assessment from the eight studies showed that four were high-quality case reports and two were moderate-high, while the two case series were limited in their methodology. As none of the studies showed a high risk of bias safeguards, the clinical descriptions, imaging confirmation, operative details, and outcomes were consistently robust (Table 4).

**Table 4:** Combined JBI appraisal summary for risk of bias assessment of the eight studies. JBI = Joanna Briggs Institute

Study	Type	JBI quality summary	Overall rating
Sammut et al. [4]	Case report	Clear patient demographics, clinical presentation, imaging, operative details, outcome; clear mechanism (phytobezoar)	High
Al Kandari et al. [5]	Case report	Well-structured. Complete clinical timeline, clear diagnostic tools, treatment, complications, and follow-up	High
Elhardello et al. [1]	Case report	clear documentation, radiology results, operative findings, and histology, which confirms ischaemic ulcer; follow-up is included	High
De Hous et al. [6]	Case report	Clinical and CT findings reported; clear mechanism (metastatic melanoma); recovery; no adverse issues	High
Gonzalez-Pezzat et al. [7]	Case report	Clear presentation, imaging, and operative description; unclear cause; limited follow-up	High-moderate
Kröll et al. [8]	Case report	Clinical and operative description; uncertain mechanism; letter format limits details	Moderate-high
Goitein et al. [2]	Case series	Good case descriptions, but lack inclusion criteria and methodological detail; strong imaging and operative findings	Moderate
Kalaiselvan et al. [3]	Case series	Detailed cases but poor methodological transparency; significant variation in outcomes; unclear inclusion strategy	Moderate-low

Discussion

Late JJ perforation is an extremely rare phenomenon, requiring a high index of suspicion among clinicians. Reported figures include 0.42% and 0.18% in the case series reported by Goitein et al. [2] and Kalaiselvan et al. [3], respectively. Therefore, it should be considered in the differential diagnosis for any patient presenting with abdominal pain and sepsis anytime after the LRYGB operation. Moreover, the JJ joint should be thoroughly assessed for the site of perforation during exploratory laparoscopy or laparotomy of such presentation, especially if no clear preoperative cause is suggested. Although patients classically present with signs of generalised peritonitis, these can sometimes be masked by the relatively high body mass index. Therefore, tachycardia might be the only reliable sign to find [3]. These might result in delayed diagnosis and management. In two of the reported cases, the patient presented to the emergency department on two separate days with pain before surgery was ultimately offered [3,7]. Although an erect chest X-ray frequently showed free air in the abdomen, this imaging modality offers very limited value compared to cross-sectional imaging with oral and intravenous contrast, which not only provides the diagnosis of a perforation but can suggest the exact location as well as associated pathology, e.g., an abscess or mass [6,7]. This can help guide the surgeon in planning the operative approach and time, as well as obtaining well-informed consent from the patient.

Several mechanisms have been hypothesised in the studied literature, including ischaemia, obstructing phytobezoar, malignancy, recent emotional stress or surgery, and the use of non-steroidal anti-inflammatory drugs (NSAIDs) [1-6]. However, the most frequent observation across reported cases was the absence of a clearly identifiable underlying cause [2,7,8]. Chronic risk factors such as smoking or *Helicobacter pylori *infection, although more relevant to marginal GJ ulcer, can play a role in JJ perforation, even though they were not seen frequently in these reported cases. The variability in the time interval between the LRYGB and this event may support the heterogeneous theory of pathogenesis. With the current limited understanding, it might be more pragmatic to divide the late JJ perforation after LRYGB into two main categories: primary, where no clear cause is found, and secondary, due to another pathology, e.g., tumour or phytobezoar.

It is difficult to suggest any preventive measures at this stage. However, the emphasis on avoiding the usual culprits, such as smoking or unwise use of NSAIDs, should remain the standard advice for patients with LRYGB. Infection with *Helicobacter pylori *should always be appropriately treated. An assessment of abdominal pain after LRYGB should ideally include assessment of any problems related to the JJ anastomosis, such as bezoar or mass, with appropriate imaging, as well as modified endoscopic tools to assess for any ulcers that can be treated before causing perforation.

Analysis of the reported JJ anastomosis technique of the original LRYGB demonstrates that late perforation at the JJ joint can occur following any of the two commonly used methods of JJ construction, including combined stapled anastomosis and hand-sewn closure of defect [1,2,7], or totally hand-sewn techniques [3]. This indicates that the perforation is not necessarily related to the technique used in joint construction at the index operation. Moreover, it reflects that no technique has been proven to be superior to the other in avoiding this serious complication, at least within smaller case studies.

Management approaches varied among reported cases. Approximately one-third (33.3%) of patients underwent laparoscopic repair [1-3], whereas the majority (66.6%) required open intervention via midline laparotomy [2-8]. This predominance of open surgery likely reflects the surgical complexity in the management of JJ perforation and the critical clinical condition of affected patients, in whom immediate open exploration is often necessary. Laparoscopic approach should be reserved for stable patients with early presentation and limited contamination, as well as the availability of surgical expertise. Otherwise, an emergency laparotomy should be the preferred life-saving procedure.

Regardless of the surgical technique adopted, the majority of repair methods were successful. The postoperative recovery can vary significantly from a few days to discharge following simple laparoscopic suture closure and wash out, to prolonged hospital admission after laparotomy, redo of anastomosis, or stoma formation. Early diagnosis and surgery are key factors in determining a patient’s recovery. The presence of associated pathology, such as a mass or an abscess, can also affect the treatment option and the subsequent outcomes. Exteriorising the bowel as a defunctioning stoma is a reasonable option for very sick patients, where a high chance of postoperative leak is expected.

One case managed with the creation of a controlled fistula using a Foley catheter by Kalaiselvan et al. [3] unfortunately resulted in the single reported mortality case in this review. Therefore, it does not seem to be an attractive treatment option in such a scenario. The presence of this mortality case clearly indicates that this is a serious, life-threatening complication, and every effort should be made to support these patients. This requires urgent multidisciplinary collaboration between emergency doctors, surgeons, anaesthetists, and intensive care specialists.

Limitations

Points limiting the strength of this review are mainly related to the paucity of data due to the rarity of this condition. Despite the overall high JBI quality of bias assessment for the eight published studies, the low level of evidence and the poor design of these studies must be acknowledged. However, this should be used to encourage surgeons to publish more cases, as this condition might be under-reported.

Future directions

It might be helpful for future practice to conduct a thorough evaluation of patients presenting with late JJ perforation for potential risk factors. Tests to rule out infective, inflammatory, or malignant bowel pathology are extremely important to gain a better understanding of this late adverse event. Peritoneal fluid should ideally be sent for microbiological assessment. Histopathological examination of any resected specimens can help clarify the underlying cause.

## Conclusions

Late JJ perforation following LRYGB is exceedingly uncommon, occurring in fewer than 1% of cases. Available data are sparse and primarily limited to individual case reports, making it difficult to draw firm conclusions about underlying causative mechanisms. Given the limited and low-quality evidence, maintaining a high index of suspicion remains essential to ensuring timely diagnosis and appropriate management. Surgeons are encouraged to report more cases to improve the knowledge about this rare, life-threatening complication.
